# Energy stress-induced circZFR enhances oxidative phosphorylation in lung adenocarcinoma via regulating alternative splicing

**DOI:** 10.1186/s13046-023-02723-z

**Published:** 2023-07-17

**Authors:** Zhifei Ma, Hao Chen, Zhijun Xia, Jing You, Chencheng Han, Siwei Wang, Wenjia Xia, Yongkang Bai, Tongyan Liu, Lin Xu, Guoren Zhou, Youtao Xu, Rong Yin

**Affiliations:** 1 Department of Thoracic Surgery, Jiangsu Key Laboratory of Molecular and Translational Cancer Research, Nanjing Medical University Affiliated Cancer Hospital & Jiangsu Cancer Hospital & Jiangsu Institute of Cancer Research, Nanjing, 21009 P.R. China; 2 Department of Thoracic Surgery, Nanjing Drum Tower Hospital Clinical College of Nanjing Medical University, Nanjing, 210008 Jiangsu China; 3 Department of Thoracic Surgery, Affiliated Tumor Hospital of Nantong University, Nantong, 226361 China; 4 Biobank of Lung Cancer, Jiangsu Biobank of Clinical Resources, Nanjing, 21009 P.R. China; 5 Collaborative Innovation Center for Cancer Personalized Medicine, Nanjing Medical University, Nanjing, 211116 P.R. China; 6 Department of Oncology, Jiangsu Cancer Hospital & the Affiliated Cancer Hospital of Nanjing Medical University & Jiangsu Institute of Cancer Research, Nanjing, 210009 Jiangsu P.R. China; 7 Department of Science and Technology, Nanjing Medical University Affiliated Cancer Hospital & Jiangsu Cancer Hospital & Jiangsu Institute of Cancer Research, Nanjing, 21009 P.R. China

**Keywords:** Energy stress, circRNA, Lung adenocarcinoma, Oxidative phosphorylation, Alternative splicing.

## Abstract

**Background:**

Circular RNAs (circRNAs) contribute to multiple biological functions and are also involved in pathological conditions such as cancer. However, the role of circRNAs in metabolic reprogramming, especially upon energy stress in lung adenocarcinoma (LUAD), remains largely unknown.

**Methods:**

Energy stress-induced circRNA was screened by circRNA profiling and glucose deprivation assays. RNA-seq, real-time cell analyzer system (RTCA) and measurement of oxygen consumption rate (OCR) were performed to explore the biological functions of circZFR in LUAD. The underlying mechanisms were investigated using circRNA pull-down, RNA immunoprecipitation, immunoprecipitation and bioinformatics analysis of alternative splicing. Clinical implications of circZFR were assessed in 92 pairs of LUAD tissues and adjacent non-tumor tissues, validated in established patient-derived tumor xenograft (PDTX) model.

**Results:**

CircZFR is induced by glucose deprivation and is significantly upregulated in LUAD compared to adjacent non-tumor tissues, enhancing oxidative phosphorylation (OXPHOS) for adaptation to energy stress. CircZFR is strongly associated with higher T stage and poor prognosis in patients with LUAD. Mechanistically, circZFR protects heterogeneous nuclear ribonucleoprotein L-like (HNRNPLL) from degradation by ubiquitination to regulate alternative splicing, such as myosin IB (MYO1B), and subsequently activates the AKT-mTOR pathway to facilitate OXPHOS.

**Conclusion:**

Our study provides new insights into the role of circRNAs in anticancer metabolic therapies and expands our understanding of alternative splicing.

**Supplementary Information:**

The online version contains supplementary material available at 10.1186/s13046-023-02723-z.

## Introduction

Lung cancer is the second most commonly diagnosed cancer and remains the leading cause of cancer-related death worldwide in 2020. [1] Lung adenocarcinoma (LUAD) is the major histological subtype of non-small cell lung cancer (NSCLC), accounting for approximately 40% of lung malignancies. [2] In recent years, rapid advances in early detection and multimodal therapy have greatly improved survival benefits. However, LUAD remains highly lethal, with an average 5-year survival rate of only 15%.^3^ Continued investigation of the underlying molecular mechanisms is required to expand the treatment options for patients with LUAD.

Rapidly growing tumors are often confronted with nutrient (e.g., glucose) and energy deficient environments. Reprogramming energy metabolism to support cell proliferation and division has been recognized as a hallmark of cancer. [3] One prominent example is the Warburg effect, wherein cancer cells metabolize glucose even in the presence of oxygen. However, emerging evidence suggests that lung cancer cells exhibit enhanced glycolysis and oxidative phosphorylation (OXPHOS). [4] The glucose concentration in tumor tissues is much lower than in normal tissues, and cancer cells under energy stress rely on OXPHOS for survival, which brings about clonal selection of a more malignant phenotype. [5, 6].

CircRNAs are generated by precursor mRNA back-splicing and modulate biological processes including cancer metabolism. [7] For example, circACC1 activates the AMPK pathway to alter cellular lipid storage in response to serum deprivation. [8] Owing to their circular structure, circRNAs are more stable than linear RNAs and hold promise as disease biomarkers. CircRNAs can function through microRNA (miRNA) sponges, interaction with various proteins, cap independent translation, and splicing regulation. [9, 10].

Alternative splicing involves the removal of introns from messenger RNA precursors, thereby generating specific functional transcript variants. [11] Splicing alterations are common in cancer and contribute to almost every hallmark, including cell growth and tumor metabolism. [12] However, the biological role of circRNAs, especially from the perspective of alternative splicing, in response to energy stress in LUAD remains largely elusive.

Herein, by performing expression profiling of circRNAs in LUAD through microarray and glucose starvation assays, we found that hsa_circ_0072088 (circZFR) was significantly upregulated in LUAD tissues and was induced upon energy stress. CircZFR, associated with poor clinical outcome, promoted OXPHOS and cell proliferation in LUAD by stabilizing the heterogeneous nuclear ribonucleoprotein L-like (HNRNPLL) protein. Furthermore, transcript splicing analysis of RNA-seq data revealed the alternative splicing switch in *MYO1B* as a functional downstream target, and *MYO1B* full-length (MYO1B-fl) transcripts activated OXPHOS via AKT-mTOR signaling. Taken together, our findings demonstrated that circZFR exerted its effect on splicing regulation to support OXPHOS for metabolic adaptation, and could be a candidate target for antitumor therapy.

## Methods

### Patient specimens

All tumors and adjacent tissues were obtained by surgical resections from patients with LUAD without preoperative treatment at the Department of Thoracic Surgery, Nanjing Medical University Affiliated Cancer Hospital (Nanjing, China). Written informed consent was obtained from all the participants. The study was approved by the Ethics Committee of the Nanjing Medical University Affiliated Cancer Hospital and was carried out in accordance with the provisions of the Ethics Committee of Nanjing Medical University.

### Cell culture and transfection

Human LUAD cells (A549, HCC827, H1975, PC9) were obtained from the Stem Cell Bank, Chinese Academy of Sciences, and tested to ensure that they were mycoplasma-free. The cell lines were cultured in RPMI-1640 medium. For the glucose-deprivation assay, cells were cultured in high (10 mM) or low (2.5 mM) glucose. All siRNA, ASO, and miRNA mimics were purchased from RiboBio (China). The cells were transfected using Lipofectamine RNAiMax (Invitrogen, USA). For the expression vector, full-length cDNA of human circZFR was cloned into the expression vector pCD5-ciR and verified by sequencing (Geneseed Biotech, China). Flag-HNRNPLL, MYO1B-fl, and MYO1B-t vectors were designed as described previously [13, 14]. For the luciferase reporter vector, the circZFR sequence was cloned into the pmirGLO vector (Promega, USA). The ATP assay kit (MAK190, Sigma-Aldrich, USA) was used for cellular ATP quantification.

### RNA analysis and RT-PCR

Total RNA was extracted using TRIzol reagent (Invitrogen). The subcellular localization assay was performed using the PARIS kit (Ambion, USA). Genomic DNA was extracted using PureLink Genomic DNA Mini kit (Invitrogen), according to the manufacturer’s protocol. For RNase R digestion, 2 µg RNA was incubated for 30 min at 37 °C with 3U/µg of RNase R (Epicentre, USA). Total RNA was reverse transcribed using the PrimeScript RT Master Mix (Takara) and amplified using PowerUp SYBR Green Master Mix (Invitrogen). *GAPDH*, *ACTB*, and *snRNA U6* were used as internal standards. Primers and oligonucleotide sequences are listed in Supplementary Table S1.

### Cell proliferation, cell cycle, and apoptosis assays

Cell proliferation was evaluated using the real-time cell analyzer system (ACEA Biosciences, USA) and EdU kit (RiboBio) following the manufacturer’s instructions. For cell cycle analysis, cells were labeled with PI/RNase Staining Buffer (BD Biosciences, USA), and the DNA content was detected by flow cytometry (FACScan, BD Biosciences). For the cell apoptosis assay, cells were double-stained with annexin V-PE (BD Biosciences) and propidium iodide (PI) (Sigma) and analyzed using a FACS scan flow cytometer.

### Oxygen consumption

Cells (8,000 cells per well) were plated in XF 96-well microplates and incubated for 24 h at 37 °C in 5% CO_2_. OCR was measured using the XF96 Extracellular Flux Analyzer (Seahorse Bioscience, USA) according to the manufacturer’s protocol.

### RNA-seq analysis

For RNA-seq of circZFR knockdown, total RNA was extracted using TRIzol reagent (Invitrogen), and RNA quality was checked using Bioanalyzer 2200. cDNA libraries were prepared using the Ion Total RNA-Seq kit v2.0 (Life Technologies). The cDNA libraries were then processed for the Proton sequencing process. For RNA-seq of circRNA overexpression, sequencing libraries were generated using the NEBNext UltraTM RNA Library Prep kit (NEB, USA) for Illumina. The samples were analyzed on the cBot Cluster Generation System using the TruSeq PE Cluster kit v4-cBot-HS (Illumina, USA). After cluster generation, the prepared libraries were sequenced on an Illumina platform and paired-end reads were generated. After removing the adaptor sequences, reads with > 5% ambiguous bases, and low-quality reads, the clean reads were then aligned to the human genome (hg38). Batch effects were removed by SVA (R package) and differential expression was calculated using edgeR (R package). Gene set enrichment analysis (GSEA, http://software.broadinstitute.org/gsea) was performed using MSigDBv6. PROGENy (R package) was used for pathway signature analysis. The RNA sequencing data has been deposited in the Gene Expression Omnibus database (GSE193064).

### RNA immunoprecipitation and circRNA pull-down assay

For the RNA immunoprecipitation assay, the EZMagna RIP kit (Millipore, USA) was used, following the manufacturer’s protocol. For the circRNA pull-down assay, biotin-labeled circZFR probes were synthesized by RiboBio and the assay was performed as described previously. [15] Briefly, cell lysates were prepared in the IP lysis buffer and pre-cleared by incubation with streptavidin beads (65,001, Invitrogen) at 4 °C for 1 h. CircRNA probes immobilized on the streptavidin beads were then added to the cell lysates and incubated overnight at 4 °C. After washing five times, the beads were boiled in SDS buffer for protein elution and MS.

### Immunoprecipitation

Cells transfected with flag-HNRNPLL vectors were lysed in IP lysis buffer with protease inhibitors. MG132 (20 µM) was added before to inhibit HNRNPLL degradation. For immunoprecipitation, antibodies against flag (CST) were added to the lysates and incubated overnight at 4 °C with rabbit IgG (5 µg) as the negative control. Pierce Protein A/G Magnetic Beads (88,802, Thermo Scientific) were added and incubated for 1 h at room temperature.

### RNA-protein interaction prediction

Analysis of the binding sites of circZFR and *HNRNPLL* was performed as described previously. [15] Briefly, we used Mfold to calculate the lowest theoretical value of free energy for circZFR and then used 3dRNA to generate the 3D structure. The 3D model of circZFR was placed in HDOCK together with HNRNPLL (UniProt, Q8WVV9) for RNA/protein interaction simulation. Two atoms (one in RNA and the other in protein) were considered to be docked with each other if their distance was < 4 Å.

### Splicing quantification

Differential alternative splicing (AS) events were analyzed using rMATS (R package). Events with |PSI (percentage spliced in) | > 0.05, p < 0.05, and FDR < 0.1 were identified as significantly differentially expressed AS events. The RNA-seq datasets (ENCSR490DYI) from ENCODE were used to analyze HNRNPLL knockdown. DARTS was used for further deep learning-based validation.

### Western blotting

Western blotting was performed as described previously, [16] using the following primary antibodies: HNRNPLL (26769-1-AP, Proteintech, USA), MYO1B (sc-393,053, Santa Cruz Biotechnology, USA), FLAG (14,793 S, Cell Signaling Technology, USA), phospho-AKT (4060, Cell Signaling Technology), phospho-mTOR (5536T, Cell Signaling Technology), and β-actin (ab6276, Abcam, UK). Relative protein levels were analyzed by ImageJ.

### In vivo tumor growth assay

Animal experiments were conducted in accordance with the Institute for Laboratory Animal Research Guide for the Care and Use of Laboratory Animals, and the protocols were approved by the Animal Committee of Nanjing Origin Biosciences. Female BALB/c nude mice (4–6 weeks old; Beijing Vital River Laboratory Animal Technology, China) were used for the xenograft model. 5 × 10^**6**^ HCC827 cells transfected with circZFR stable overexpression or control vectors were suspended and injected into the flanks of mice. For the PDTX model, primary lung adenocarcinoma tissue samples were split into approximately 2 mm [17] pieces and directly implanted into the subcutaneous space. When the tumor reached approximately 200 mm [17] in size, mice were randomly divided into two experimental groups and received intratumoral injection of 10 nM negative control or ASO targeting circZFR twice per week for four weeks. After the mice were sacrificed, the tumors were weighed and processed for further histological analysis.

### Statistical analysis

Results are presented as mean ± standard deviation of the mean. Statistical analyses were performed using the SPSS 25 software (Abbott Laboratories, USA). The differences between two groups were assessed by Student’s t-test, and comparisons among three or more groups were first assessed by one-way analysis of variance (ANOVA), as indicated in the figure legends. The cut-off values for survival analysis were determined using the maxstat (R package). Results with a p value 0.05 or less were considered statistically significant.

## Results

### CircZFR is an upregulated circRNA in LUAD in response to energy stress

**Fig. 1:**
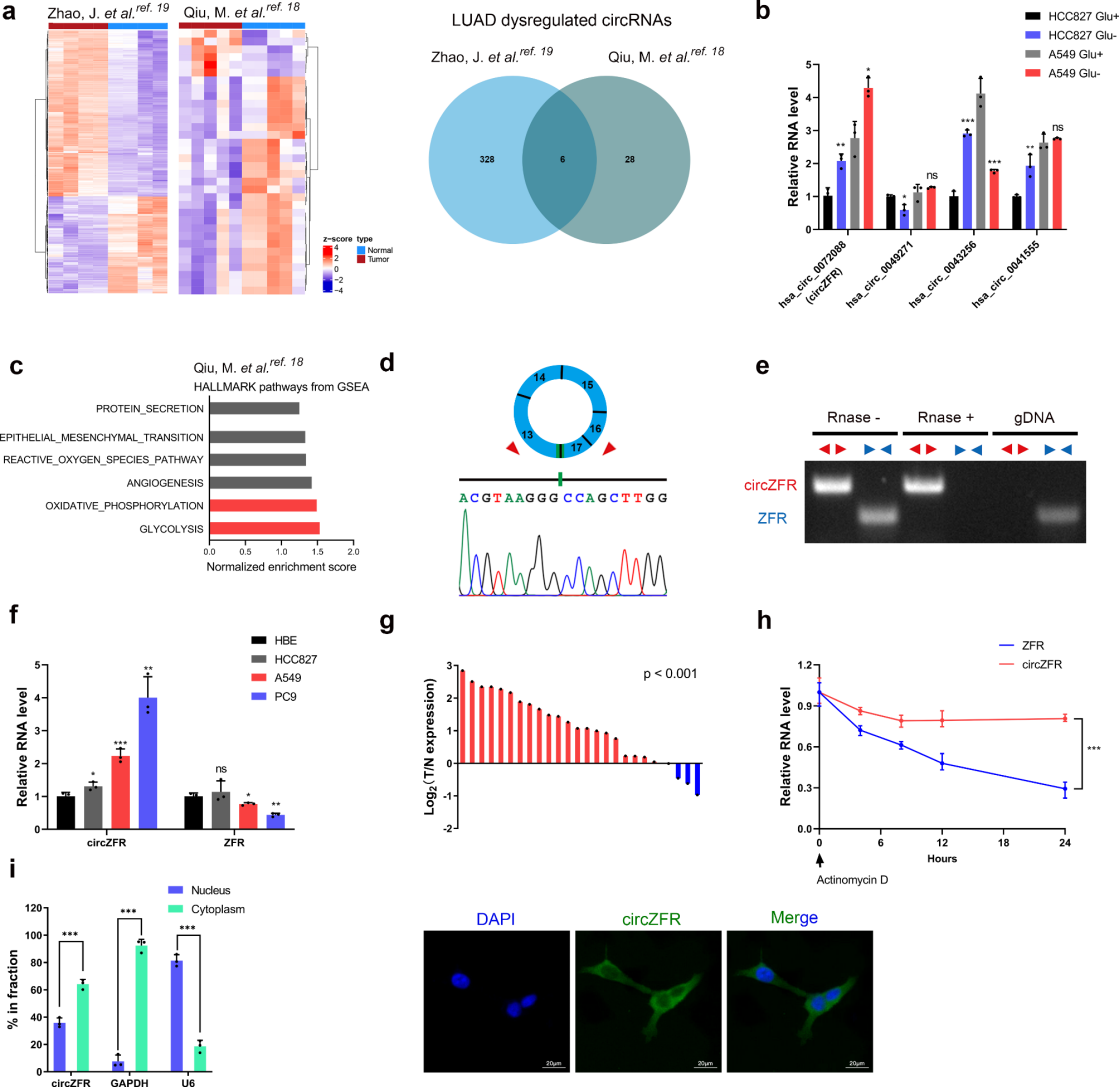
Characterization of circZFR as a circular RNA in response to glucose starvation in LUAD. **a** Heatmap of differentially expressed circRNAs between LUAD tissues and adjacent nontumor tissues in microarrays. (GSE101586, |log(Fold change)| > 1.5, p < 0.05; GSE1016840, |log (Fold change) | > 1, adjust.p < 0.05). Gene expression in z score-transformed value was shown (left). Pie chart showing dysregulated circRNAs in both microarrays (right). **b** Expression of candidate circRNAs in response to glucose limitation. Cells were cultured in high (10mM) or low (2.5mM) glucose. **c** Top 6 hallmark pathways from GSEA enriched in patients with high circZFR expression. **d** The genomic loci and validation of circZFR by Sanger sequencing. **e** Divergent and convergent primers were designed and the gene expression with or without RNase R treatment was detected by gel electrophoresis. gDNA, genomic DNA. **f** circZFR and *ZFR* mRNA expression in LUAD cell lines and human bronchial epithelioid cells. **g** Expression of circZFR in 25 paired LUAD samples were detected by RT-PCR. *ACTB* was used as a loading control. T tumor tissue, N nontumorous tissue. **h** RNA stability of circZFR and *ZFR*. **i** RT-PCR analysis of subcellular fractionation in the A549 cells (left). Confocal microscopy images of circZFR (green) in A549 cells (right). Nuclei was stained with DAPI (blue). Scale bars, 20 μm. Data are shown as mean ± SD. *p < 0.05, **p < 0.01, ***p < 0.001, two-tailed Student’s t test

To screen for dysregulated energy stress-related circRNAs in lung adenocarcinoma (LUAD), we first analyzed our previous circRNA microarray data (GSE101586) and another circRNA expression profile (GSE1016840) in paired LUAD and adjacent normal tissues (Fig. 1a, left; Supplementary Fig. 1a, b). [18, 19] Unsupervised principal component analysis (PCA) performed on these datasets demonstrated that the tumor and normal groups were clearly separated (Supplementary Fig. 1c). In total, six significantly dysregulated circRNAs were identified (Fig. 1a, right). Focusing on energy stress-inducible circRNA, we further detected circRNA expression in LUAD cell lines by real-time polymerase chain reaction (RT-PCR) after glucose deprivation. After hsa_circ_0029426 and hsa_circ_0000662 were excluded due to nonspecific amplification, only hsa_circ_0072088 levels were significantly increased in both glucose-limited cell lines (Fig. 1b). Integrated analysis of microarray-derived circRNA and mRNA expression profiles (GSE101586) revealed that gene sets related to glycolysis and oxidative phosphorylation were significantly enriched in patients with high hsa_circ_0072088 expression (Fig. 1c). Moreover, hsa_circ_0072088 levels were positively correlated with starvation-related genes (Supplementary Fig. 1d).

Circularized by exon 13–17 of *ZFR* with a length of 693nt according to circBase (http://www.circbase.org), hsa_circ_0072088 was termed circZFR. The back-spliced junction of circZFR was confirmed by Sanger sequencing (Fig. 1d). RNase R assay verified that circZFR was resistant to digestion. In contrast, bands generated by convergent primers specifically amplifying linear *ZFR* mRNA disappeared after RNase R treatment (Fig. 1e). The genomic structure showed that circZFR was flanked by two introns containing Alu elements that facilitated the generation of circRNA (Supplementary Fig. 1e). [20] The exon sequences of circZFR, instead of reverse complementary intronic Alu elements, are highly conserved, hence the existence of circZFR was not detected in mouse cell lines (Supplementary Fig. 1f). To validate the microarray analysis, we assessed circZFR expression in LUAD cell lines and tissues. RT-PCR results revealed that circZFR, but not *ZFR*, was significantly upregulated in LUAD cell lines compared to human normal bronchial epithelial cell line (Fig. 1f). Consistently, circZFR RNA levels were increased in LUAD tissues (Fig. 1g; Supplementary Fig. 1g). Based on the data, HCC827 and A549 cells were selected for subsequent assays. Moreover, circZFR was more stable than *ZFR*, and its increased accumulation could be the consequence of energy stress (Fig. 1h). Cell fractionation PCR showed that circZFR was present in both cytoplasm and nucleus, as confirmed by fluorescence in situ hybridization (FISH) assays (Fig. 1i; Supplementary Fig. 1h). These results indicate that circZFR is a glucose starvation-induced circRNA that is aberrantly expressed in LUAD.

### CircZFR promotes OXPHOS to enhance cell proliferation in LUAD

**Fig. 2:**
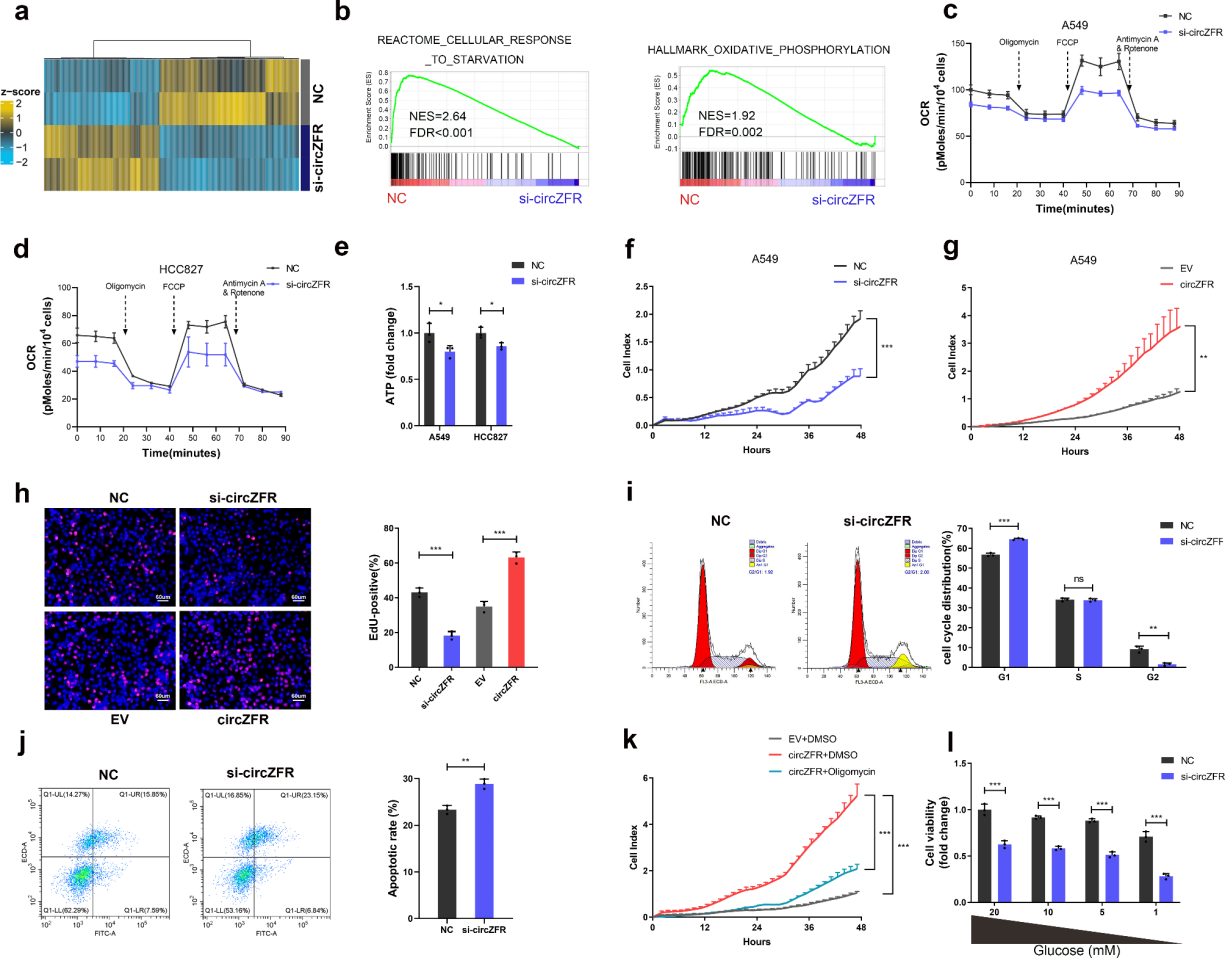
CircZFR is an oncogenic circRNA and promotes OXPHOS in vitro. **a** Heatmap of genes affected by circZFR identified using RNA-seq. **b** GSEA results used to identify the differential gene profiles. **c-e** Oxygen consumption rate (OCR) upon cells (**c, d**) and cellular ATP levels (**e**) were measured after transfecting with control or circZFR siRNA in A549 and HCC827. **f-j** CircZFR facilitated the proliferation of A549 cells shown by the RTCA (**f, g**), EdU (**h**), cell cycle (**i**) and apoptosis (**j**) assays. Cell numbers were determined using the ImageJ program. **k** Effect of oligomycin (200 nM) on A549 cell vitality detected by RTCA. **l** Cell viability of A549 cells treated as in (**l**). Data are shown as mean ± SD (n = 3) or typical photographs of one representative experiment. Similar results were obtained in three independent experiments. *p < 0.05, **p < 0.01, ***p < 0.001, ns, nonsignificant, two-tailed Student’s t test

Small interfering RNA (siRNA) specifically targeting the junction was designed to investigate the function of circZFR. The siRNA effectively knocked down circZFR, whereas no detectable effects on *ZFR* expression were detected in HCC827 and A549 cells (Supplementary Fig. 2a). In contrast, the overexpression vector remarkably improved circZFR levels, whereas *ZFR* expression showed no obvious changes (Supplementary Fig. 2b). To elucidate the role of circZFR in LUAD, RNA-seq was performed and a total of 671 dysregulated genes (FDR < 0.05, |logFC| > 1) were detected after silencing circZFR in A549 cells (Fig. 2a, Supplementary Table S2). Gene set enrichment analysis (GSEA) revealed that biological processes, such as “cellular response to starvation”, “oxidative phosphorylation (OXPHOS)”, and “cell cycle” were significantly enriched in the circZFR-affected genes (Fig. 2b; Supplementary Fig. 2c). OXPHOS is considered as the major process for optimal proliferation under glucose-deprived conditions. [21] Therefore, we hypothesized that circZFR induced by energy stress might directly promote OXPHOS in LUAD cells, which was subsequently validated by measuring the oxygen consumption rate (OCR). Knockdown of circZFR reduced the OCR in HCC827 and A549 cells (Fig. 2c, d). ATP is mainly generated by glucose via glycolysis or OXPHOS in cancer cells. [22] Cellular ATP levels were also repressed after circZFR downregulation (Fig. 2e). In gain-of-function assay, circZFR overexpression remarkably improved the OCR (Supplementary Fig. 2d) as well as ATP production (Supplementary Fig. 2e) in both LUAD cell lines.

The proliferation ability of HCC827 and A549 cells was significantly impaired after circZFR silencing as observed by real-time cell analyzer system (RTCA) and 5-ethynyl2’-deoxyuridine (EdU) proliferation assay (Fig. 2f-h). Conversely, ectopic expression of circZFR promoted cell viability (Supplementary Fig. 2f). Flow cytometry analysis showed that circZFR knockdown induced G1 phase cell cycle arrest and apoptosis (Fig. 2i, j; Supplementary Fig. 2g, h). Transwell and Matrigel assays revealed that circZFR enhanced the migration and invasion of A549 cells (Supplementary Fig. 2i, j). Additionally, oligomycin (an inhibitor of ATP synthase to block OXPHOS) treatment repressed circZFR-induced cell proliferation, and LUAD cells under glucose limitation were more sensitive to circZFR knockdown (Fig. 2k, l). Both loss-of-function and gain-of-function assays in vitro demonstrate that circZFR is a stress-induced circRNA that regulates OXPHOS to adapt to energy shortage in LUAD.

### CircZFR interacts with HNRNPLL

**Fig. 3:**
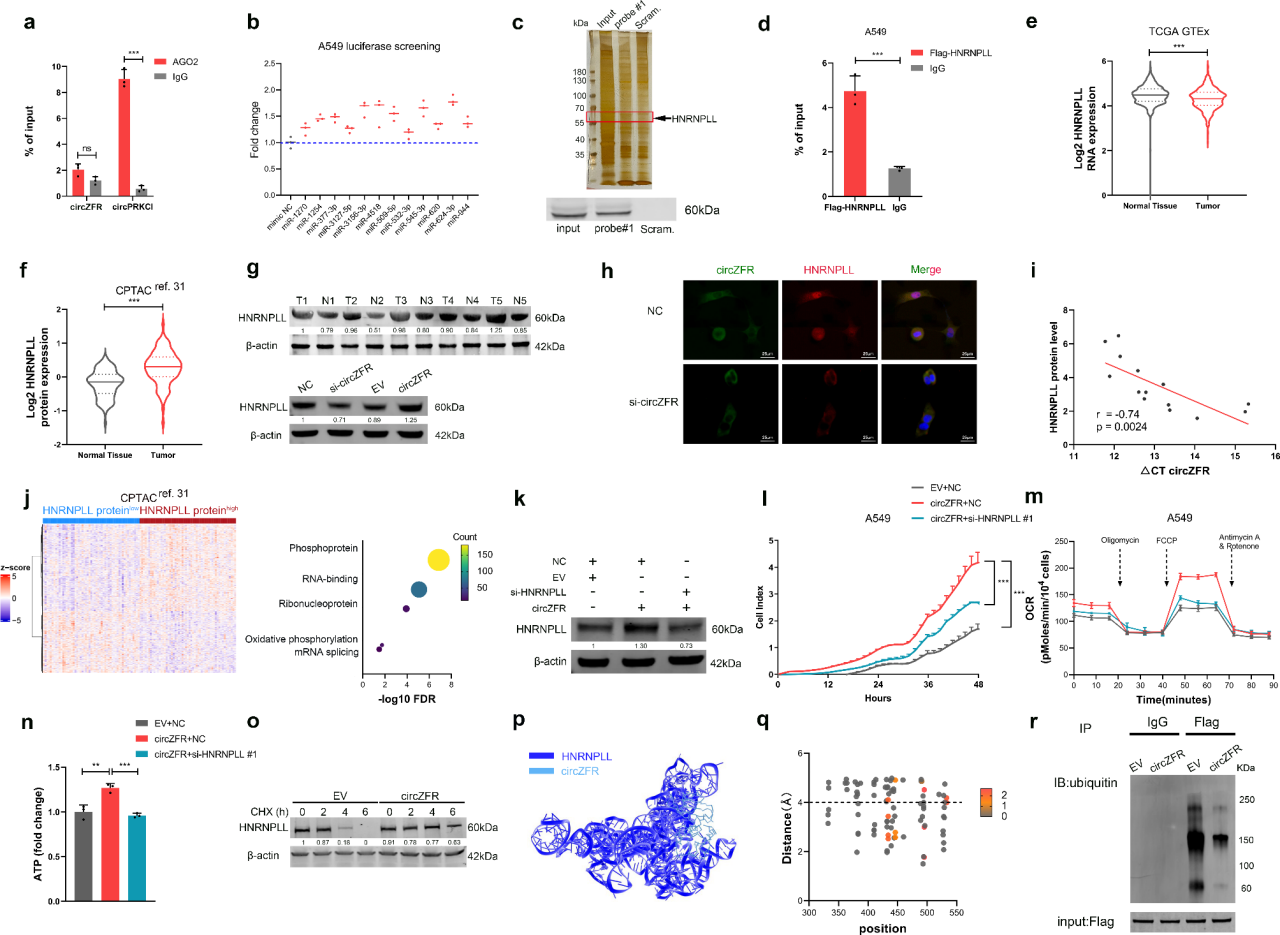
CircZFR stabilizes HNRNPLL to promote OXPHOS. **a** The enrichment of circRNAs was detected by RIP in A549 cells. circPRKCI was used as positive control. **b** Luciferase activity of circZFR transfected with candidate miRNA mimics. **c** Silver staining (upper panel) and western blotting (lower panel) of proteins retrieved by circZFR probe. Scramble probe was used as a negative control. **d** RT-PCR analysis of circZFR enriched by Flag-HNRNPLL proteins. **e** The *HNRNPLL* RNA levels in LUAD and normal lung tissues. **f** The HNRNPLL protein levels in LUAD and adjacent tissues from CPTAC cohort. **g** The HNRNPLL protein expression in lung tissues (upper panel) and the changes affected by circZFR (lower panel). **h** The fluorescence images of circZFR (green) and HNRNPLL (red) in A549 cells. Nuclei was stained with DAPI (blue). Scale bars, 25 μm. **i** Correlation analysis on circZFR RNA and HNRNPLL protein levels in LUAD tissues. ΔCT values were normalized according to *ACTB*. HNRNPLL protein levels was determined by western blotting and normalized according to β-actin. **j** Heatmap of differentially expressed genes (left) and top 5 biological processes from GO enriched (right) in the HNRNPLL protein^high^ patient group. **k-n** HNRNPLL protein expression (**k**), cell proliferation (**l**), OCR levels (**m**) and ATP levels (**n**) in A549 cells transfected with circZFR vector alone or co-transfected with HNRNPLL siRNA. **o** The expression kinetics of HNRNPLL in A549 cells treated with CHX and circZFR expression vector. **p** The predicted 3D model of circZFR and HNRNPLL by HDOCK software. **q** Ubiquitination site prediction and docking capability determined by distance on circZFR. **r** HNRNPLL ubiquitination treated with circZFR expression vector was assayed by immunoprecipitation. Data are shown as mean ± SD (n = 3) or typical photographs of one representative experiment. Similar results were obtained in three independent experiments. *p < 0.05, **p < 0.01, ***p < 0.001, two-tailed Student’s t test

Due to the absence of long enough open reading frame and evidence from ribosome profiling analysis of circZFR (data not shown), [23] we eliminated the possibility that circZFR functioned by generating functional peptides. Recent studies have shown that majority of circRNAs act as miRNA sponges to modulate gene expression and circZFR has been reported to promote cancer progression based on ceRNA mechanism. [24, 25] However, AGO2 RNA immunoprecipitation assays (RIP) demonstrated that AGO2 did not recruit circZFR (Fig. 3a). After identifying the target miRNAs of circZFR using prediction tools, [26–28] we analyzed tumor suppressor miRNAs in LUAD from The Cancer Genome Atlas (TCGA) database and published literature (Supplementary Fig. 3a). Finally, 12 potential miRNAs were selected for luciferase reporter assays; however, none remarkably repressed the luciferase activity of circZFR (Fig. 3b; Supplementary Fig. 3b). These results strongly indicated that miRNA sponges might not be the predominant mechanism of circZFR. Hence, we designed biotin-labeled junction-specific probes and performed circRNA pull-down assay in combination with mass spectrometry (MS) to identify circZFR-interacting proteins. The most abundant protein retrieved by the circZFR probe was HNRNPLL, readily visualized by sensitive silver staining, and further confirmed by western blotting (Fig. 3c; Supplementary Fig. 3c). Flag-HNRNPLL RIP assay using A549 and HCC827 cell lysates also validated the interaction between circZFR and HNRNPLL (Fig. 3d; Supplementary Fig. 3d).

### CircZFR stabilizes HNRNPLL protein to facilitate the progression of LUAD

Splicing factors such as hnRNP proteins regulate metabolic switch and play key roles in tumorigenesis, but the role of *HNRNPLL* in LUAD remains largely unknown. [29] We examined its expression in four independent cohorts of patients with LUAD (n = 1165) and observed reduced *HNRNPLL* RNA levels in tumor tissues compared to normal tissues (Fig. 3e; Supplementary Fig. 3e). [30–32] Conversely, the protein levels were elevated in tumor tissues and further confirmed by western blotting (Fig. 3f, g; Supplementary Fig. 3f). Since the RNA expression of *HNRNPLL* was irrelevant to its protein expression in tumor tissues (r = -0.17, FDR = 0.25; Supplementary Fig. 3g), we hypothesized that circZFR, upregulated in LUAD, might participate in the post-transcriptional regulation of *HNRNPLL*. Silencing of circZFR in both LUAD cell lines significantly reduced HNRNPLL protein levels without affecting the mRNA expression and vice versa (Fig. 3g, lower panel; Supplementary Fig. 3h, i). FISH results demonstrated that circZFR was relatively co-localized with HNRNPLL, and silencing of circZFR suppressed HNRNPLL protein expression, especially in the nucleus (Fig. 3h). The positive association between circZFR and HNRNPLL protein levels was also validated in LUAD tissues (Fig. 3i).

In support of the idea that *HNRNPLL* mediates the biological function of circZFR, we analyzed differentially expressed genes in the HNRNPLL protein^high^ and protein^low^ patients from the CPTAC cohort (Fig. 3j, left). Gene Ontology analysis showed enrichment, including OXPHOS and mRNA splicing, in patients with high levels of HNRNPLL protein expression(Fig. 3j, right). Kaplan–Meier survival analysis revealed that high levels of HNRNPLL protein indicated poor clinical outcomes (Supplementary Fig. 3j). Effective knockdown of *HNRNPLL* repressed cell proliferation (Supplementary Fig. 3k, l). Furthermore, silencing of *HNRNPLL* significantly reduced the elevated levels of protein expression, cell viability, OXPHOS, and ATP production after circZFR overexpression (Fig. 3k-n).

Since the increased HNRNPLL protein levels induced by circZFR were attributed to the prolonged half-life (Fig. 3o), we built a circZFR-HNRNPLL interaction model using distance-based approach to uncover the effect of circZFR on HNRNPLL protein stability (Fig. 3p). Several potential binding sites were predicted to be modified by ubiquitination (434 A, 446 A, 495 A, and 533 A, Fig. 3q), suggesting that circZFR could cover the ubiquitination sites of HNRNPLL to prevent protein degradation. As expected, we observed a significant decrease in ubiquitinated HNRNPLL after ectopic expression of circZFR, whereas circZFR vectors with a mutation in the binding sites abolished the effect on HNRNPLL ubiquitination and protein expression (Fig. 3r; Supplementary Fig. 3m, n). Thus, we conclude that circZFR enhances HNRNPLL protein stability by blocking ubiquitination and thereby elevated HNRNPLL protein levels mediate OXPHOS and tumor growth in LUAD.

### CircZFR modulates alternative splicing via *HNRNPLL*

**Fig. 4:**
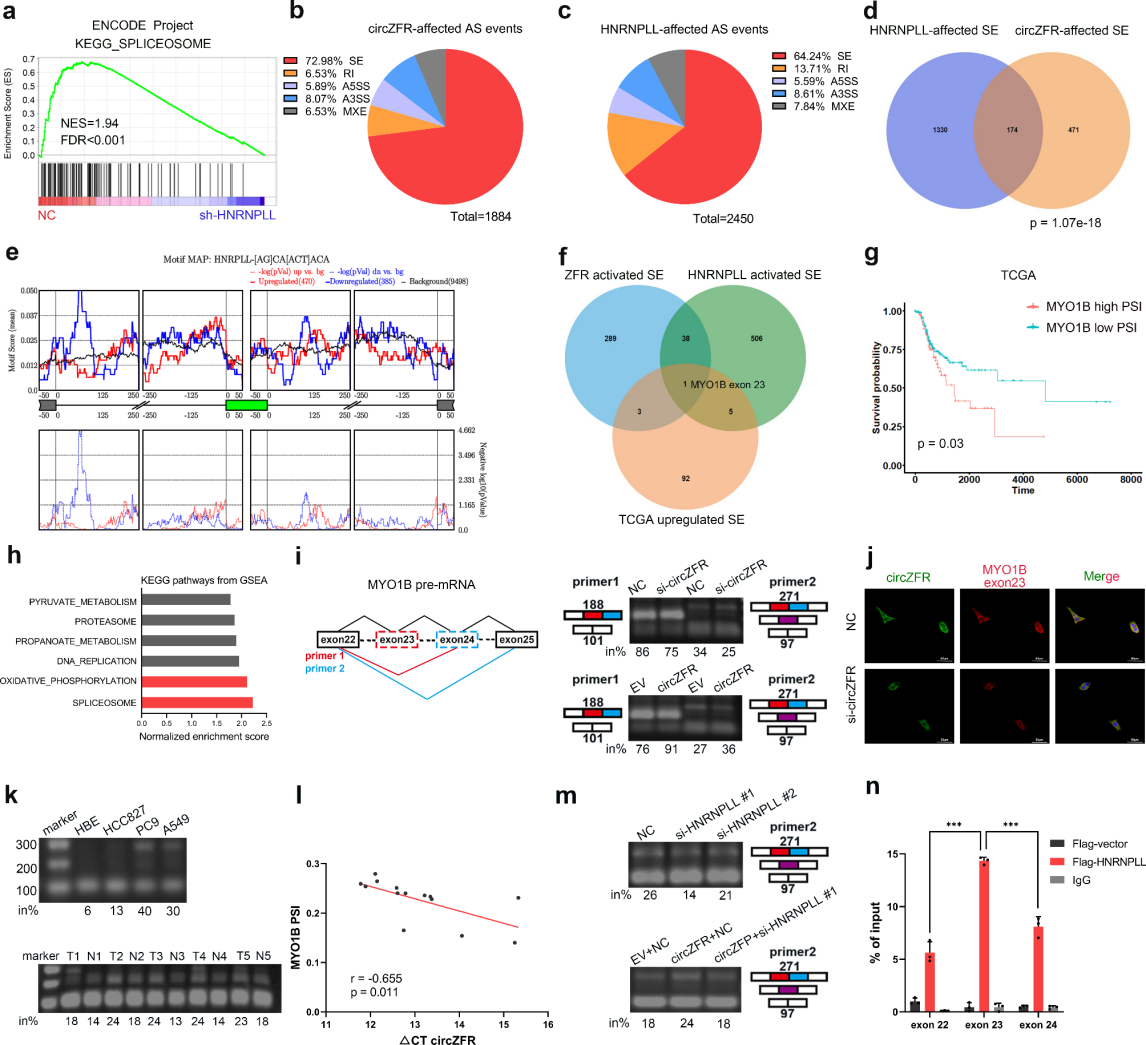
The effects of circZFR and *HNRNPLL* on alternative splicing of *MYO1B*. **a** GSEA results of the differential genes affected by *HNRNPLL* knockdown. **b, c** Distribution of alternative splicing events affected by circZFR (**b**) and *HNRNPLL* (**c**). SE skipped exon, RI retained intron, A5SS alternative 5’ splice site, A3SS alternative 3’ splice site, MXE mutually exclusive exons. **d** Venn diagram illustrating the overlap of altered exon skipping events upon circZFR overexpression or *HNRNPLL* depleting. P value was calculated by χ2 test. **e** HNRNPLL binding site analyses on alternatively spliced exons. **f** Pie chart showing the screening of potential functional target of both circZFR and *HNRNPLL.***g** Kaplan-Meier analysis of the DFI of the LUAD patients. **h** Top 6 hallmark pathways from GSEA enriched in patients with high *MYO1B* PSI scores from TCGA project. **i** Left: Diagram of the splicing variants of *MYO1B* mRNA and the primers for RT-PCR detection of exon 23 (primer 1) and exons 23 and 24 (primer 2) inclusion/exclusion. Right: RT-PCR validation of *MYO1B* exons upon knockdown or overexpression of circZFR in A549 cells. in%, percentage of inclusion. Red, exon 23, blue, exon 24, purple, exon 23/24. **j** The fluorescence images of circZFR (green) and *MYO1B* exon23 (red). Nuclei was stained with DAPI (blue). Scale bars, 50 μm. **k** Splicing pattern of *MYO1B* in LUAD cell lines (upper panel) and tissues (lower panel) as detected by RT-PCR. T, tumor tissue, N, nontumorous tissue. **l** Correlation analysis on circZFR and *MYO1B* PSI in LUAD tissues. **m** The effects of *HNRNPLL* on *MYO1B* exons in A549 cells. **n** Interaction of *MYO1B* exons and HNRNPLL examined by RT-PCR with RIP assays in A549 cells. Data are shown as mean ± SD (n = 3). Similar results were obtained in three independent experiments or typical photographs of one representative experiment. *p < 0.05, **p < 0.01, ***p < 0.001, two-tailed Student’s t test

It is reported that *HNRNPLL* induces T-cell activation by regulating alternative splicing. [13] A closely related protein, HNRNPL, has also been observed to facilitate tumorigenic capacity by controlling caspase-9 pre-mRNA processing in NSCLC. [33] RNA-seq analysis using shRNA against *HNRNPLL*, from the ENCODE project, revealed statistically enriched pathways including RNA splicing and cell cycle (Fig. 4a; Supplementary Fig. 4a). [34] To examine the role of circZFR in alternative splicing, we performed high-depth RNA-seq (> 30X) after circZFR overexpression in HCC827 cells (Supplementary Fig. 4b). [35] PCA on the RNA-seq data of circZFR knockdown and overexpression confirmed that the phenotypes observed above were on-target effects of circZFR (Supplementary Fig. 4c). Analysis of transcript splicing identified 1884 and 2450 splicing events that were significantly altered after circZFR overexpression and *HNRNPLL* knockdown, respectively (|PSI| > 0.05 and FDR < 0.1). We observed that exon skip events represent the majority (72.98% and 62.24%), which were further confirmed by deep-learning-based predictions (Fig. 4b, c; Supplementary Fig. 4d). Since high incidence of exon skip events, especially in LUAD, has been observed previously, we focused on these most promising alternative splicing events and found a significant overlap between skipped exons (SE) affected by circZFR and *HNRNPLL*, including *BPTF*, a reported target of *HNRNPLL* (Fig. 4d). [11, 36] Gene Ontology analysis of these overlapping genes revealed biological processes associated with alternative splicing and phosphoprotein (Supplementary Fig. 4e). Additionally, we noted a significant enrichment of *HNRNPLL* binding motifs around the introns of transcripts affected by circZFR, and the motifs in 5′ of cassette exons tended to promote their inclusion (Fig. 4e), in line with previous PAR-CLIP results. [37, 38] The data suggests that circZFR and *HNRNPLL* share a substantial number of downstream target SE and in general, have consistent effects on alternative splicing.

To explore the functional target SE in LUAD, we observed the differential expression patterns of SE from TCGA datasets and noted exon 23 of *MYO1B* (myosin IB) as an inclusive exon in LUAD induced by both circZFR and *HNRNPLL* (Fig. 4f; Supplementary Fig. 4f-h). [11] Increased exon 23 inclusion was related to shorter survival time in LUAD (Fig. 4g). Both spliceosome and OXPHOS were significantly enriched in patients with high *MYO1B* PSI (percentage spliced in) scores and therefore we chose *MYO1B* as a potential target (Fig. 4h). The human *MYO1B* gene (NM_001130158) has 31 exons, of which exons 23 and 24 are subject to splicing regulation. RT-PCR using primers for exon 23 (primer 1) and exons 23 and 24 (primer 2) revealed that circZFR could promote exon 23 inclusion of *MYO1B* (Fig. 4i; Supplementary Fig. 4i). We then chose primer 2, which could amplify all exon-including or -skipping isoforms of *MYO1B*, for subsequent studies. FISH assay revealed that circZFR knockdown decreased MYO1B exon 23 levels (Fig. 4j). As expected, the transcript variants with exon 23 inclusion were increased in both LUAD cells and tissues, corresponding to circZFR expression (Fig. 4k, l; Supplementary Fig. 4j). Furthermore, *HNRNPLL* knockdown greatly decreased the inclusion levels of exon 23 and almost reversed the effect of circZFR overexpression (Fig. 4m; Supplementary Fig. 4k). To test the association between HNRNPLL and *MYO1B* transcripts, we performed RIP assay and observed approximately two-fold enrichment of exon 23 compared to adjacent exons (Fig. 4n; Supplementary Fig. 4l). Collectively, circZFR can regulate alternative splicing and facilitate *MYO1B* exon 23 inclusion via *HNRNPLL*.

### *MYO1B* splicing is a functional target of circZFR via AKT-mTOR signaling

**Fig. 5:**
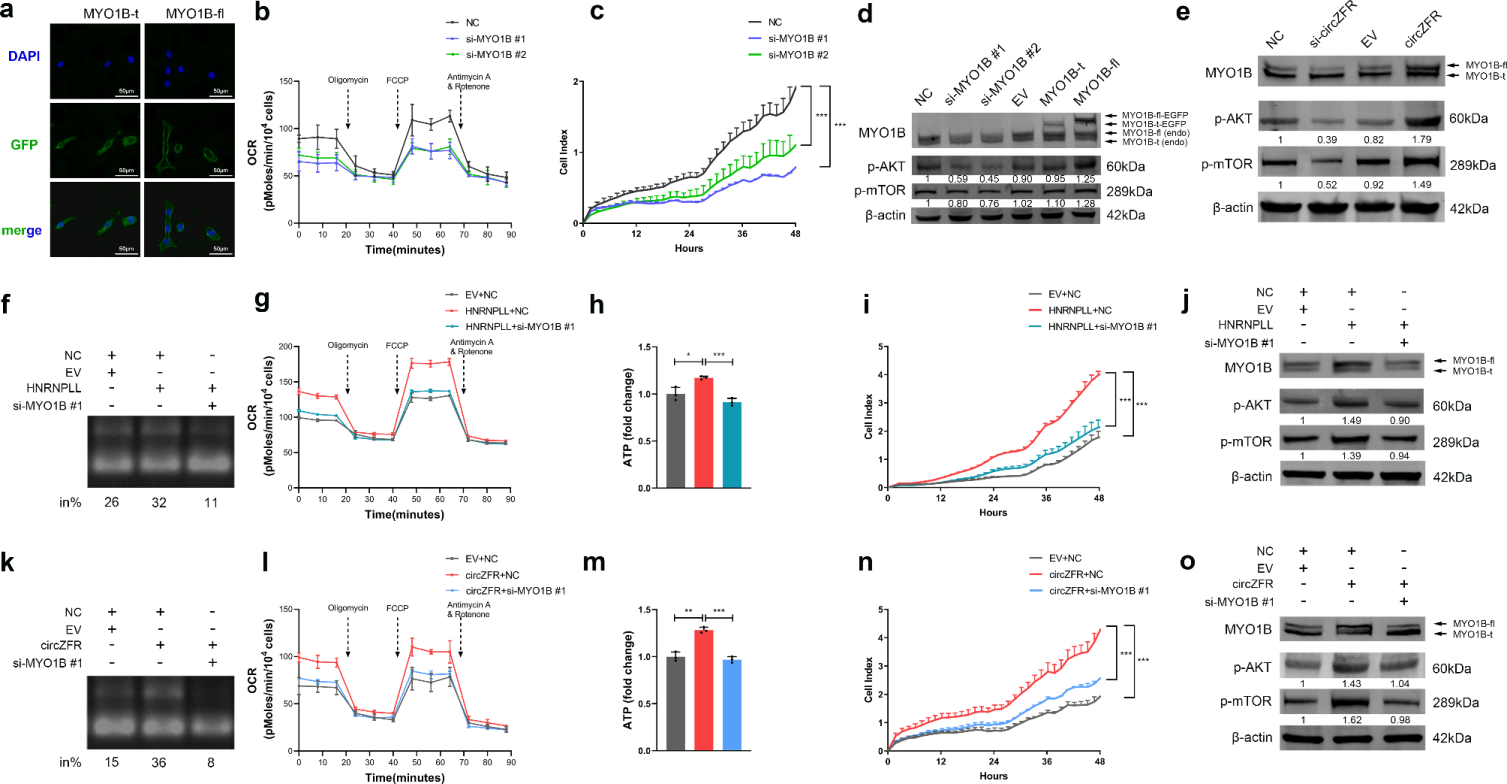
MYO1B-fl partially recapitulates the biological function of circZFR via AKT-mTOR signaling. **a** Subcellular distribution of different *MYO1B* isoforms. Nuclei was stained with DAPI (blue). Scale bars: 50 μm. **b, c** The changes of OCR levels (**b**) and cell vitality (**c**) in A549 cells after transfecting with control or MYO1B-fl siRNAs. **d** Western blot of the indicated proteins in the extracts of A549 cells. **e** The protein levels of MYO1B-fl, phospho-AKT and phospho-mTOR induced by circZFR in A549 cells. **f-j***MYO1B* exons (**f**), OCR levels (**g**), cellular ATP (**h**), cell proliferation (**i**) and protein expression (**j**) in A549 cells transfected with HNRNPLL vector alone or co-transfected with *MYO1B* siRNA. **k-o***MYO1B* exons (**k**), OCR levels (**l**), cellular ATP (**m**), cell proliferation (**n**) and protein expression (**o**) in A549 cells transfected with circZFR vector alone or co-transfected with *MYO1B* siRNA. Data are shown as mean ± SD (n = 3). Similar results were obtained in three independent experiments or typical photographs of one representative experiment. *p < 0.05, **p < 0.01, ***p < 0.001, ANOVA followed by Tukey’s test

During metabolic adaptation, AKT-mTOR signaling, the fundamental regulator of cancer metabolism and stress response, is induced to protect cells from death due to OXPHOS in various tumor types. [39, 40] As shown in Fig. 4i-k, *MYO1B* full-length transcripts containing both exons 23 and 24 (MYO1B full-length transcripts) were the dominant form (approximately 80%) of transcripts including exon 23. A previous study has shown that membrane-localized MYO1B full-length transcripts promote gliomagenesis via the AKT pathway. [14] Considering that AKT-mTORC1 signaling was significantly enriched in circZFR, *HNRNPLL*, and MYO1B-fl-associated genes (Supplementary Fig. 5a, b), we constructed full-length and truncated MYO1B EGFP-vectors (named MYO1B-fl and MYO1B-t, respectively) to validate whether the AKT-mTORC1 pathway serves as a downstream target. FISH assay showed that MYO1B-fl was preferentially located in the cytomembrane, whereas MYO1B-t was distributed in the cytoplasm (Fig. 5a). Knockdown of MYO1B-fl inhibited OXPHOS and cell viability (Fig. 5b, c; Supplementary Fig. 5c-e). Ectopic expression of MYO1B-fl, but not MYO1B-t, increased AKT and mTOR phosphorylation, which were repressed by siRNA targeting MYO1B exon 23 (Fig. 5d), suggesting that MYO1B isoforms differ in subcellular localization and MYO1B-fl promotes LUAD progression. CircZFR, therefore, might serve as an activator of AKT-mTORC1 signaling under glucose deprivation by modulating MYO1B splicing. We then verified that circZFR induced MYO1B-fl, phospho-AKT, and phospho-mTOR protein expression (Fig. 5e; Supplementary Fig. 5f). Rescue experiments revealed that silencing MYO1B-fl significantly repressed the increased MYO1B exon 23 inclusion, OXPHOS rates, cellular ATP levels, cell proliferation, MYO1B-fl protein expression, AKT-mTORC1 phosphorylation levels, and migration after *HNRNPLL* or circZFR overexpression (Fig. 5f-o; Supplementary Fig. 5g, h). Taken together, we demonstrate that circZFR promotes *MYO1B* exon 23 inclusion to regulate OXPHOS and enhance tumor progression through AKT-mTORC1 signaling. Additionally, knockdown of circZFR, *HNRNPLL*, and MYO1B-fl, which were all induced by glucose limitation, markedly impaired tolerance to energy stress, emphasizing the dependence of the circZFR-HNRNPLL-MYO1B-fl axis in the harsh environment of LUAD (Supplementary Fig. 5i, j).

### CircZFR expression correlates with poor clinical prognosis

**Fig. 6:**
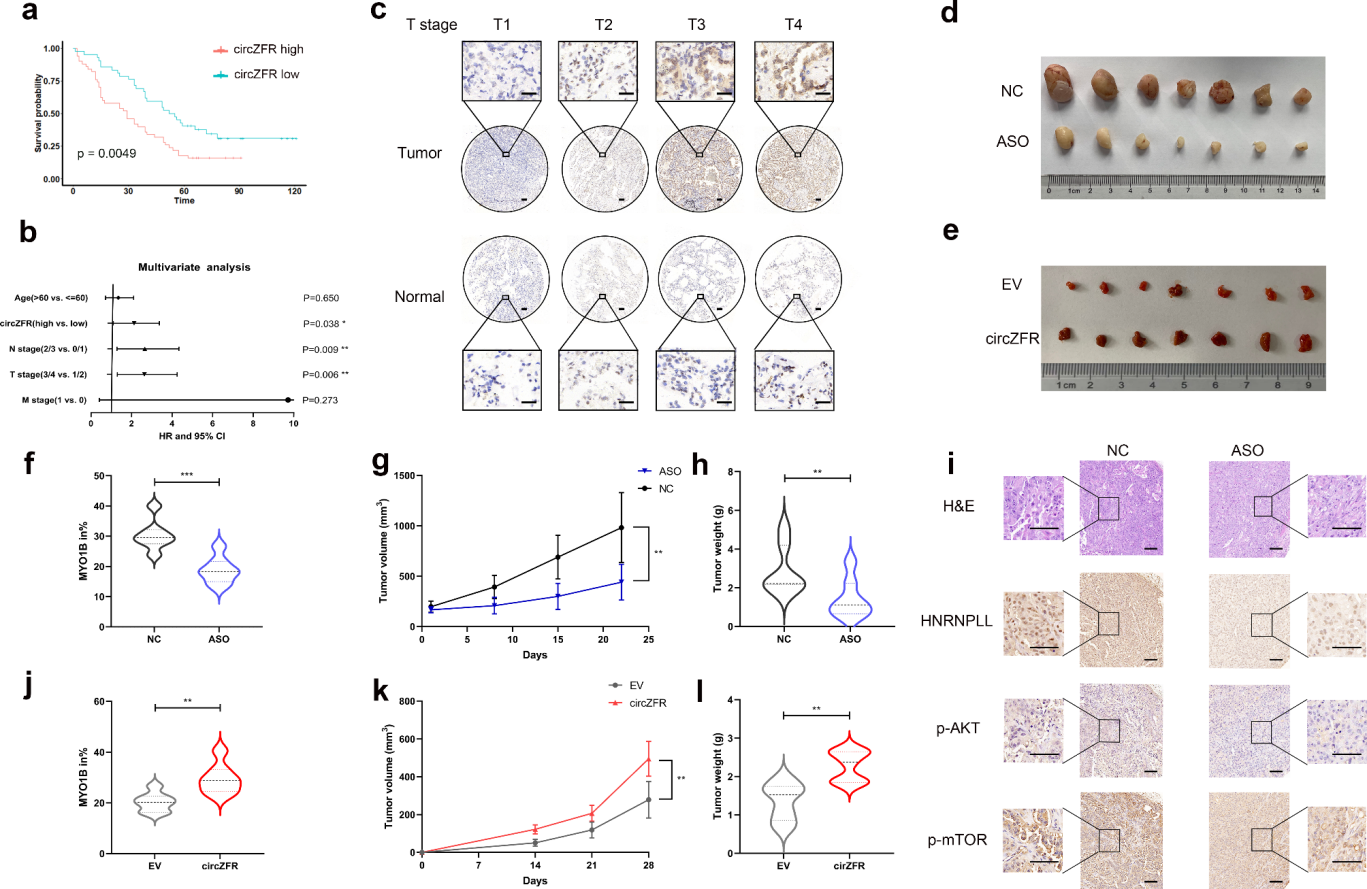
CircZFR promotes tumor growth in vivo. **a** Survival was analyzed and compared between patients with high and low levels of circZFR. **b** Multivariable analysis of circZFR in LUAD TMA. **c** The expression of circZFR analyzed by CISH in TMA was correlated with T stage. Scale bar indicates 100 μm. **d, e** Representative data of xenograft tumors isolated from PDTX models (**d**) and tumors in nude mice-bearing HCC827 cells (**e**). **f**-**h** The inclusion levels of *MYO1B* exon23 (**f**), volumes (**g**) and weights (**h**) in xenograft tumors isolated from PDTX models. (n = 7 mice per group). **i** Multi-label IHC staining of H&E, HNRNPLL, phospho-AKT and phospho-mTOR. Scale bar indicates 100 μm. **j-l** The inclusion levels of *MYO1B* exon23 (**j**), volumes (**k**) and weights (**l**) of subcutaneous xenograft tumors isolated from nude mice (n = 7 mice per group). Data are shown as mean ± SD (n = 7). *p < 0.05, **p < 0.01, two-tailed Student’s t test

We next performed a tissue microarray (TMA) using 92 pairs of LUAD tissues and adjacent non-tumor tissues to explore the clinical relevance of circZFR. Kaplan–Meier survival analysis demonstrated that patients with LUAD with high circZFR immunoreactive scores had a significantly shorter overall survival time (HR = 1.93, p = 0.0049; Fig. 6a). Univariate and multivariate regression analyses revealed that circZFR expression was an independent prognostic factor in patients with LUAD (HR = 2.07, p = 0.038; Fig. 6b; Supplementary Fig. 6a). Additionally, we found that circZFR levels were upregulated in tumors and positively correlated with higher T stage (Fig. 6c; Supplementary Fig. 6b, c). Collectively, circZFR is highly expressed in LUAD tissues, contributing to poor outcomes.

To validate the biological function of circZFR in vivo, we injected circZFR antisense oligonucleotide (ASO) into the established patient-derived tumor xenograft (PDTX) model and observed that ASO targeting circZFR significantly attenuated tumor growth as well as the inclusion of *MYO1B* exon 23 (Fig. 6d, f-h). Multi-label immunohistochemistry (IHC) showed that tumor tissues injected with ASO exhibited fewer *HNRNPLL*, phospho-AKT, and phospho-mTOR positive cells (Fig. 6i). In contrast, tumors with stable overexpression of circZFR grew more rapidly and had increased inclusion of *MYO1B* exon 23 (Fig. 6e, j-l). The preclinical models suggest that circZFR could be a potential therapeutic target for LUAD.

## Discussion

Previous studies have demonstrated that circRNAs play an important role in cancer metabolism; however, their potential involvement in energy stress is poorly defined, particularly in lung adenocarcinoma. [41] By performing microarray analysis and glucose-deprivation experiments, we identified that circZFR was upregulated in LUAD and induced by glucose limitation. GSEA analysis of circZFR-affected genes indicated significantly enriched processes in OXPHOS and the cell cycle, further validated by both gain-of-function and loss-of-function assays. Mechanistically, circZFR stabilized the HNRNPLL protein to regulate alternative splicing, such as *MYO1B*. The full-length transcripts of *MYO1B* stimulated AKT-mTOR signaling to facilitate OXPHOS and supported cell proliferation under energy stress. RT-PCR and chromogenic in situ hybridization (CISH) results suggested that circZFR expression was increased in tumors and acted as an independent prognostic factor in patients with LUAD. The identification that circZFR serves an oncogenic role by modulating alternative splicing to regulate OXPHOS is a breakthrough in cancer research.

In contrast to the classical Warburg effect that malignant tissues switch from oxidative metabolism to glycolysis even in the presence of oxygen, recent studies have demonstrated that non-small cell lung tumors enhance both glycolysis and OXPHOS simultaneously, relative to adjacent normal tissues, to promote lung cancer progression. [4, 42] During tumor development, energy deficiency exerts selective pressure and cancer cells rely on OXPHOS to adapt to energy stress, emphasizing the role of OXPHOS in metabolic reprogramming. [6, 43] Previous studies on glucose adaptation mainly focused on changes in glycolysis, [44] but our RNA-seq data and OCR assay results revealed that OXPHOS, promoted by circZFR, enhanced cell vitality. Mechanistically, AKT-mTORC1 signaling was evaluated as a downstream effector by RNA-seq, western blotting, and IHC. Although AKT signaling has been considered to facilitate aerobic glycolysis in cancer cells, abnormal activation of the AKT pathway can induce mTORC1 to promote OXPHOS for the challenge of glucose limitation, in addition to stimulating glucose transporters. [40, 45, 46] To date, the only known circRNA that modifies OXPHOS is circNFATC3 in breast and ovarian cancer cells; however, the mechanism is unknown. [47] Our study provides new insights into the regulation of OXPHOS by circRNAs to affect tumor progression.

Proteogenomic studies of LUAD reveal that only 22% of the proteins exhibit significant positive correlations with the corresponding RNA. [32] In the present study, we observed an opposite pattern of expression between *HNRNPLL* RNA and protein in LUAD. Further immunoprecipitation assays showed that circZFR protected HNRNPLL from ubiquitination-mediated protein degradation, and thus, tumor-upregulated circRNAs could function as stabilizers of oncoproteins, expanding the horizon of post-transcriptional regulation in cancer. Additionally, enrichment analysis of genes whose RNA levels are poorly related to corresponding protein levels shows processes including OXPHOS and spliceosome, [32] in line with our results of *HNRNPLL*.

Various tumors are highly dependent on aberrant splicing for cell survival, and several circRNAs play key roles in the regulation of alternative splicing. For example, circURI1 modulates RNA splicing by sequestering hnRNPM in gastric cancer. [48] Here, we identified that the splicing factor *HNRNPLL* bound to circZFR, as observed by circRNA pull-down and RIP assays. *HNRNPLL* has been reported to be a critical regulator of CD45 alternative splicing, and our study expands its role in LUAD. [13] Joint alternative splicing analysis of RNA-seq revealed significant overlap with circZFR-affected and HNRNPLL-affected exon skipping events, although the RNA-seq data of *HNRNPLL* from ENCODE dataset was obtained from HepG2 hepatocellular carcinoma cells. Furthermore, motif analysis of circZFR-affected exon skipping events demonstrated enriched *HNRNPLL* target sites located preferentially in the 5′ of cassette exons and tended to facilitate their inclusion, consistent with recent findings. [37].

Therapeutic strategies based on metabolic alterations in cancer are attracting increasing attention. However, clinical trials targeting glycolysis have failed to reduce tumor growth, [49] highlighting the metabolic plasticity by which tumor cells switch from glycolysis to OXPHOS to adapt to metabolic challenges. In our study, injection of ASO targeting circZFR in the established PDTX model significantly reduced tumor size mediated by AKT-mTOR signaling, revealing the therapeutic potential of OXPHOS inhibitors. A previous study revealed that *ZFR* was a PI3K pathway independent survival gene in breast cancer. [50] Thus, drugs targeting the exons of circZFR to repress both *ZFR* and circZFR simultaneously could be a simple and effective strategy to inhibit tumor growth.

## Conclusions

**Fig. 7:**
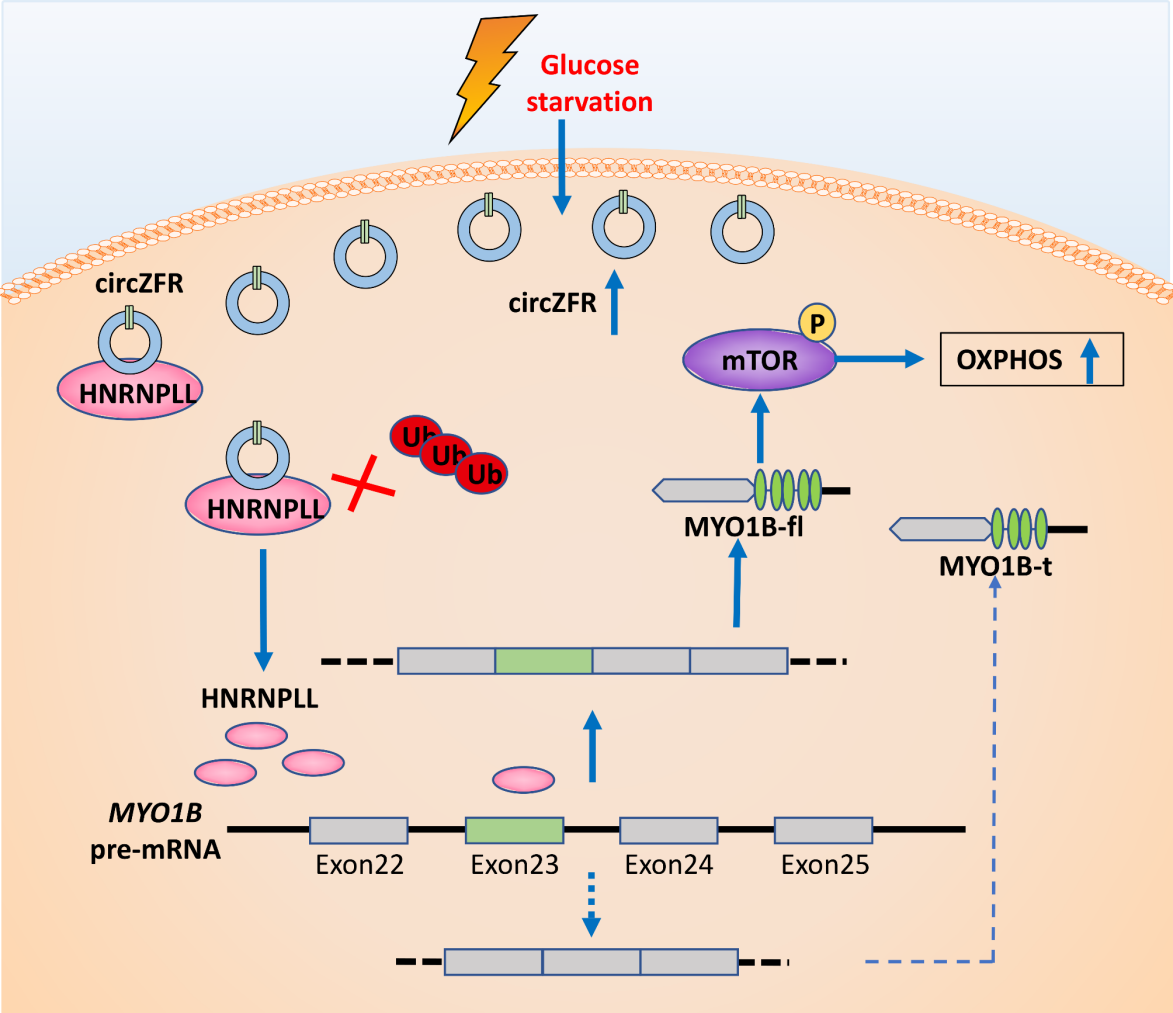
Graphical illustration of mechanism in LUAD progress. The elevated circZFR levels upon glucose stress protect HNRNPLL protein from degradation by ubiquitination, which promotes the inclusion of *MYO1B* exon 23 to enhance OXPHOS via AKT-mTOR signaling to promote tumor growth

Our results uncovered circZFR as a novel regulator of OXPHOS for adaptation to energy stress. CircZFR promoted OXPHOS and cell proliferation by stabilizing the HNRNPLL protein to modulate alternative splicing such as *MYO1B*, resulting in the activation of the AKT-mTOR pathway (Fig. 7). The study sharpens the understanding of metabolic reprogramming in cancer and allows the development of potential drug targets for LUAD.

## Electronic supplementary material

Below is the link to the electronic supplementary material.

Additional file 1: Supplementary Figure 1-6.docx

Additional file 2: Supplementary Table S1-S2.xlsx

## Data Availability

The sequence data used in this study are publicly available in Gene Expression Omnibus (GEO) at GSE193064, GSE101586, GSE1016840 and GSE140343. The data from The Cancer Genome Atlas project that were analyzed in this study were obtained at https://gdc.cancer.gov/. The RNA-seq data after shRNA-mediated knockdown of *HNRNPLL* (accession no. ENCSR490DYI) were available at ENCODE (https://www.encodeproject.org/).

## Abbreviations

CircRNA: Circular RNA
HNRNPLL: Heterogeneous nuclear ribonucleoprotein L-like
LUAD: Lung adenocarcinoma
MYO1B: Myosin IB
NSCLC: Non-small cell lung cancer
OXPHOS: Oxidative phosphorylation
