# Correction: Energy stress-induced circZFR enhances oxidative phosphorylation in lung adenocarcinoma via regulating alternative splicing

**DOI:** 10.1186/s13046-023-02771-5

**Published:** 2023-07-25

**Authors:** Zhifei Ma, Hao Chen, Zhijun Xia, Jing You, Chencheng Han, Siwei Wang, Wenjia Xia, Yongkang Bai, Tongyan Liu, Lin Xu, Guoren Zhou, Youtao Xu, Rong Yin

**Affiliations:** 1 Department of Thoracic Surgery, Jiangsu Key Laboratory of Molecular and Translational Cancer Research, Nanjing Medical University Affiliated Cancer Hospital & Jiangsu Cancer Hospital & Jiangsu Institute of Cancer Research, 21009 Nanjing, P.R. China; 2 Department of Thoracic Surgery, Nanjing Drum Tower Hospital Clinical College of Nanjing Medical University, Nanjing, 210008 Jiangsu China; 3 Department of Thoracic Surgery, Affiliated Tumor Hospital of Nantong University, Nantong, 226361 China; 4 Biobank of Lung Cancer, Jiangsu Biobank of Clinical Resources, 21009 Nanjing, P.R. China; 5 Collaborative Innovation Center for Cancer Personalized Medicine, Nanjing Medical University, 211116 Nanjing, P.R. China; 6 Department of Oncology, Jiangsu Cancer Hospital & the Affiliated Cancer Hospital of Nanjing Medical University & Jiangsu Institute of Cancer Research, 210009 Nanjing, Jiangsu P.R. China; 7 Department of Science and Technology, Nanjing Medical University Affiliated Cancer Hospital & Jiangsu Cancer Hospital & Jiangsu Institute of Cancer Research, Nanjing, 21009 People’s Republic of China

**Correction:** ***J Exp Clin Cancer Res*** **42, 169 (2023)**

**https://doi.org/10.1186/s13046-023-02723-z**

Following publication of the original article [1], an error was identified in the author list. Authors, Youtao Xu and Rong Yin were not captured as corresponding authors. The corresponding authors are:

Guoren Zhou^6*^, Youtao Xu^1*^ and Rong Yin^1,4,5,7*^

The correction do not affect the overall Conclusion of the article. The original article has been corrected.
