# Supplementary material for: Energy stress-induced circZFR enhances oxidative phosphorylation in lung adenocarcinoma via regulating alternative splicing
Source: J Exp Clin Cancer Res. 2023 Jul 17;42:169. doi: 10.1186/s13046-023-02723-z (PMC10351155; doi:10.1186/s13046-023-02723-z)
Supplement: Supplementary file 1 — Additional file 1: Supplementary Figure 1-6.docx [file 13046_2023_2723_MOESM1_ESM.docx]

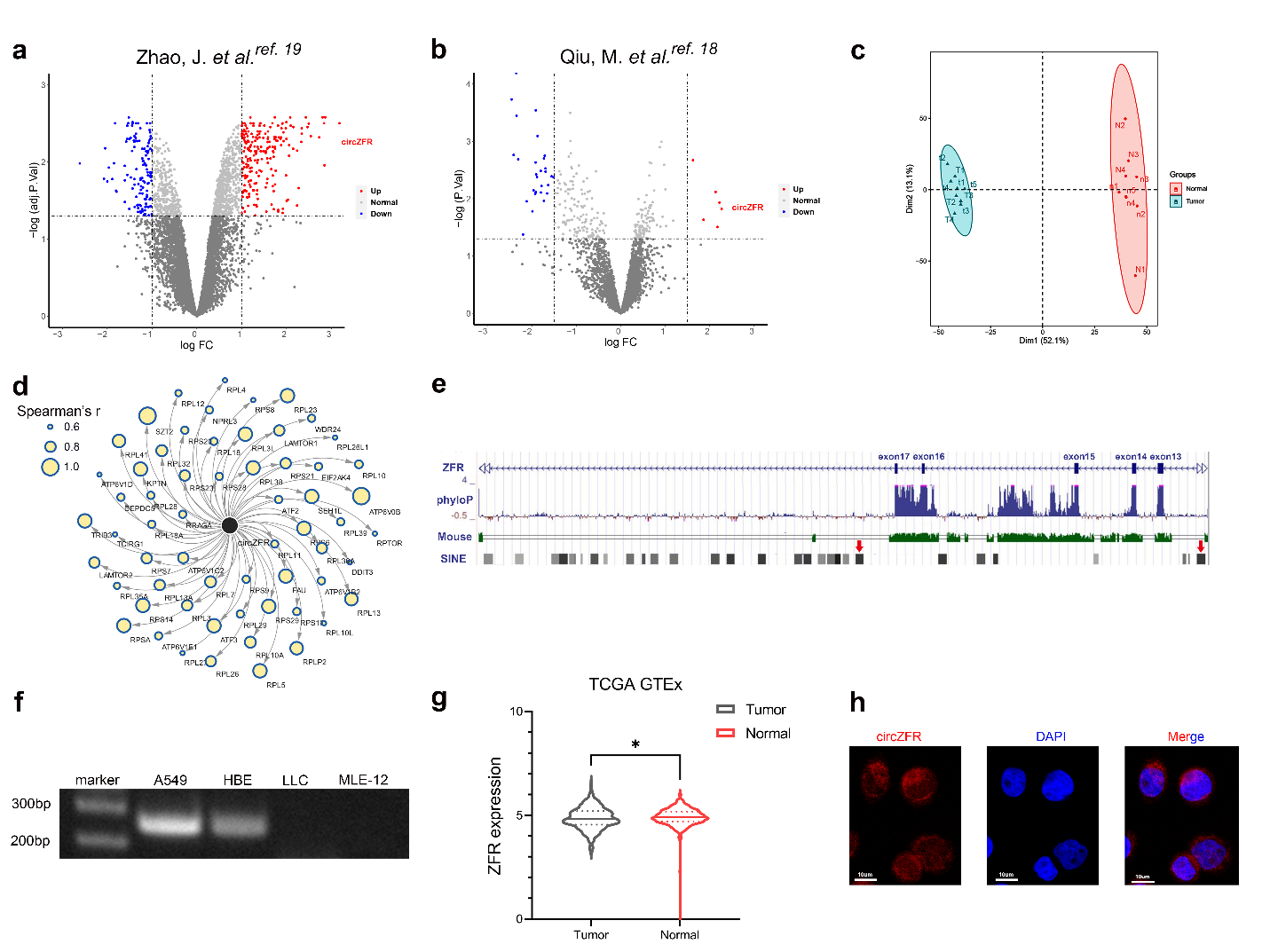


**Supplementary Figure 1. Characterization of circZFR.**

**a, b** Volcano plot showed dysregulated circRNAs in two circRNA microarrays. **c** PCA of circRNA microarrays. Ellipses represent 95% confidence regions. **d** Co-expression network of circZFR with starvation related genes defined by REACTOME_CELLULAR_RESPONSE_TO_ STARVATION gene set. A round node represented a protein-coding gene. Lines between two nodes indicate interactions between two genes. **e** Homology of *ZFR* across species from the UCSC database. SINE short interspersed nuclear elements. **f** circZFR expression in human and mouse cell lines detected by electrophoresis. **g** Confocal microscopy images of circZFR (red) in HCC827 cells. Nuclei was stained with DAPI (blue). Scale bars, 10 μm. **h** Expression levels of *ZFR* in TCGA and GTEx datasets. Data are shown as mean ± SD. *p < 0.05, Wilcox test.


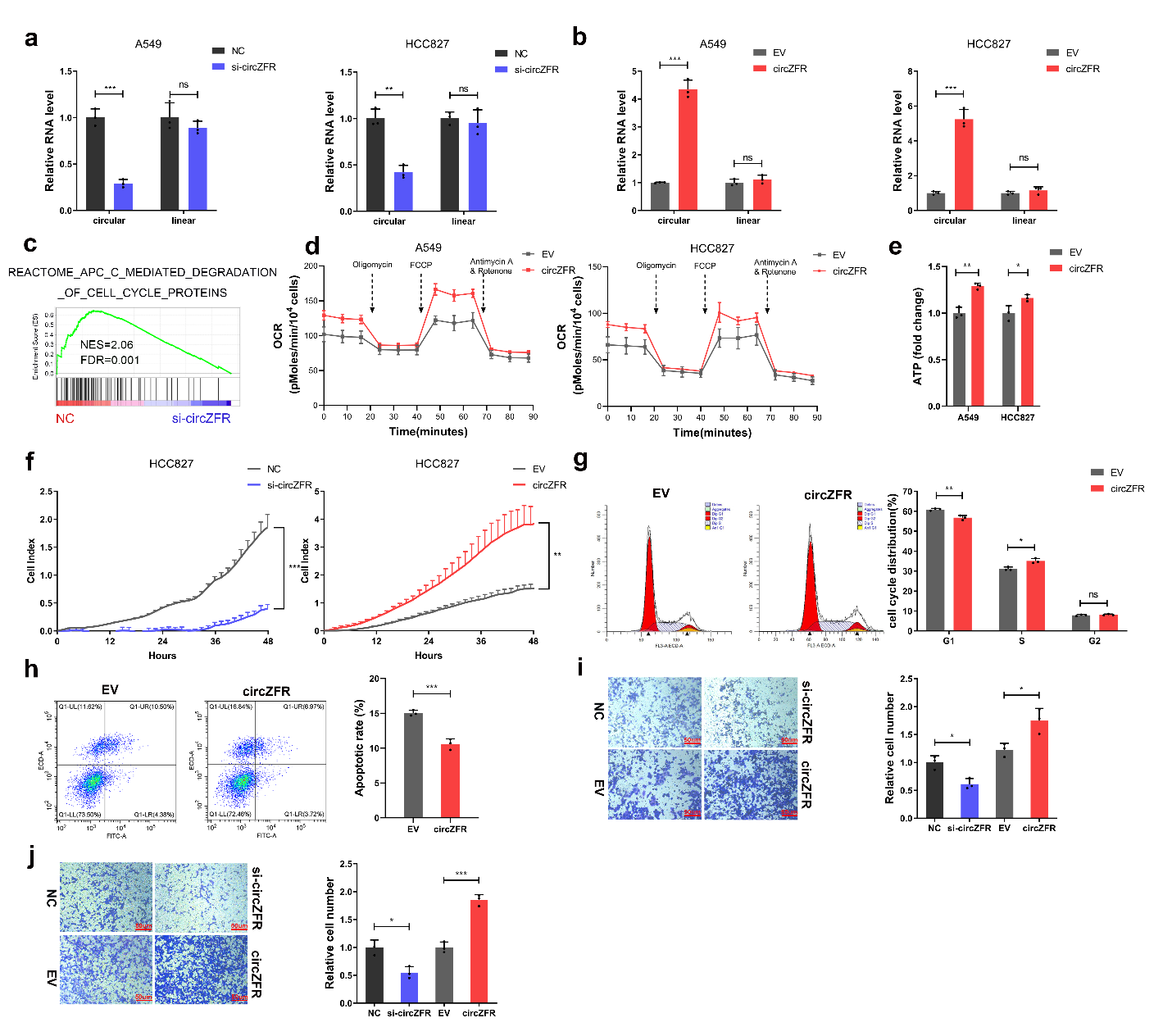


**Supplementary Figure 2. CircZFR facilitates the malignant progression of LUAD cells.**

**a, b** The efficiencies of siRNA (**a**) and expression vector (**b**) in A549 and HCC827 cells were determined by RT-PCR. **c** GSEA results of the differential genes affected by circZFR knockdown. **d, e** Oxygen consumption rate (OCR) upon cells (**d**) and cellular ATP levels (**e**) were measured after transfecting with empty vector or circZFR expression vector in A549 and HCC827. **f-h** CircZFR facilitated the proliferation shown by the RTCA (**f**), cell cycle (**g**) and apoptosis (**h**) assays. **i, j** Transwell (**i**) and matrigel (**j**) assays in A549 cells. Scale bars, 50 μm. Data are shown as mean ± SD (n=3) or typical photographs of one representative experiment. Similar results were obtained in three independent experiments. **p* < 0.05, ***p* < 0.01, ****p* < 0.001, ns, nonsignificant, two-tailed Student’s t test.


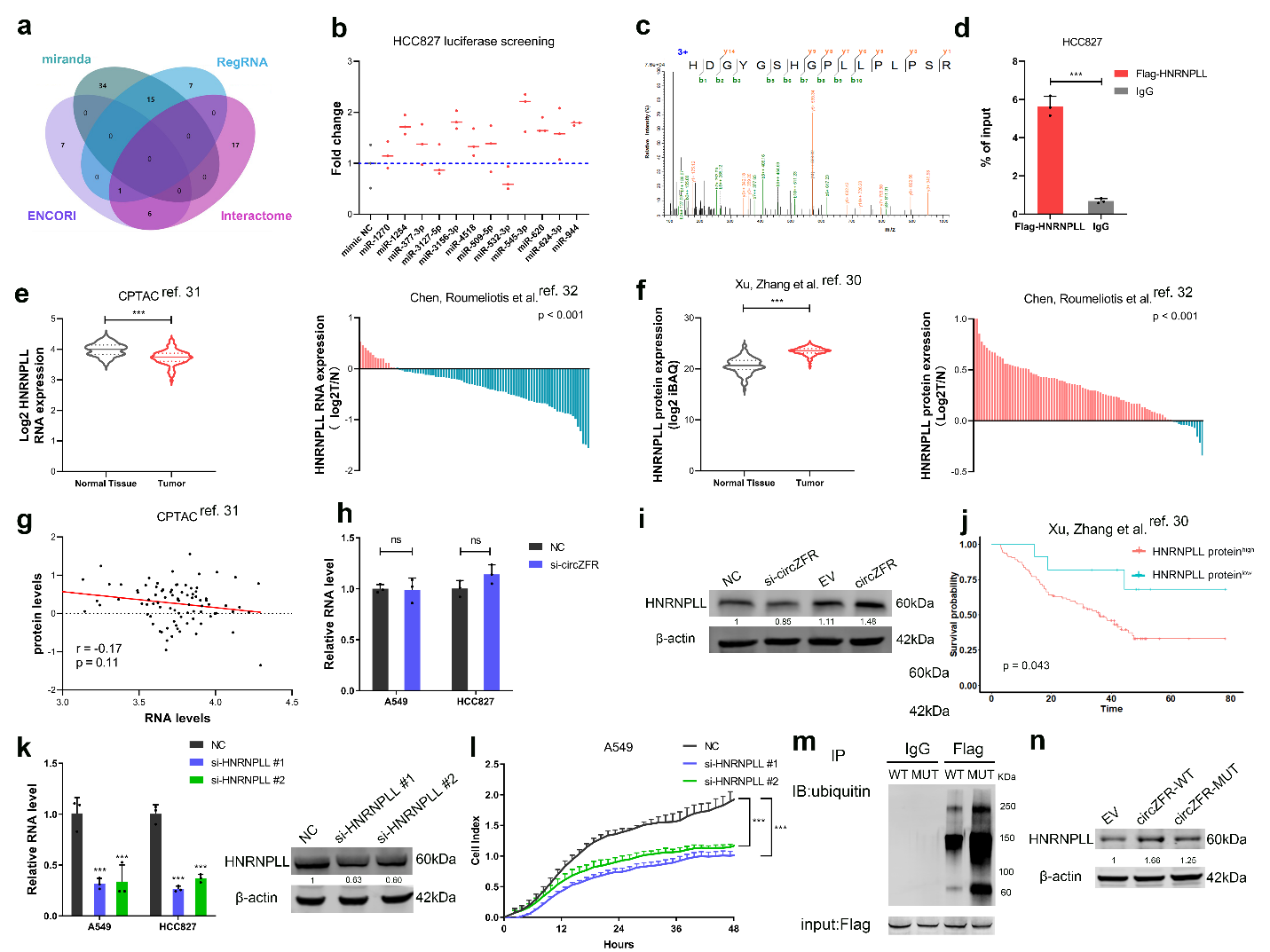


**Supplementary Figure 3. CircZFR interacts with HNRNPLL protein**

**a** Screening for miRNA candidates of circZFR using various prediction tools. **b** Luciferase activity of circZFR in HCC827 cells transfected with candidate miRNA mimics. **c** Mass spectrometry analysis for the peptides of circZFR-interacting HNRNPLL. **d** RT-PCR analysis of circZFR enriched by HNRNPLL proteins in HCC827 cells. **e, f** The HNRNPLL RNA (**e**) and protein levels (**f**) in LUAD and normal lung tissues in several LUAD cohorts. **g** Correlation plot of the RNA and protein levels of HNRNPLL in CPTAC cohort. **h, i** HNRNPLL RNA (**h**) and protein expression (**i**) affected by circZFR. **j** Kaplan-Meier analysis of the DFS of the LUAD patients. **k** The HNRNPLL RNA (left) and protein levels (right) in A549 cells transfected with HNRNPLL siRNAs. **l** Cell proliferation affected by HNRNPLL detected by RTCA. **m** The effect of circZFR-HNRNPLL binding sites mutations on HNRNPLL ubiquitination. **n** HNRNPLL protein expression in A549 cells transfected with circZFR expression vector or circZFR mutant vector. Data are shown as mean ± SD (n=3) or typical photographs of one representative experiment. Similar results were obtained in three independent experiments. **p* < 0.05, ***p* < 0.01, ****p* < 0.001, two-tailed Student’s t test.


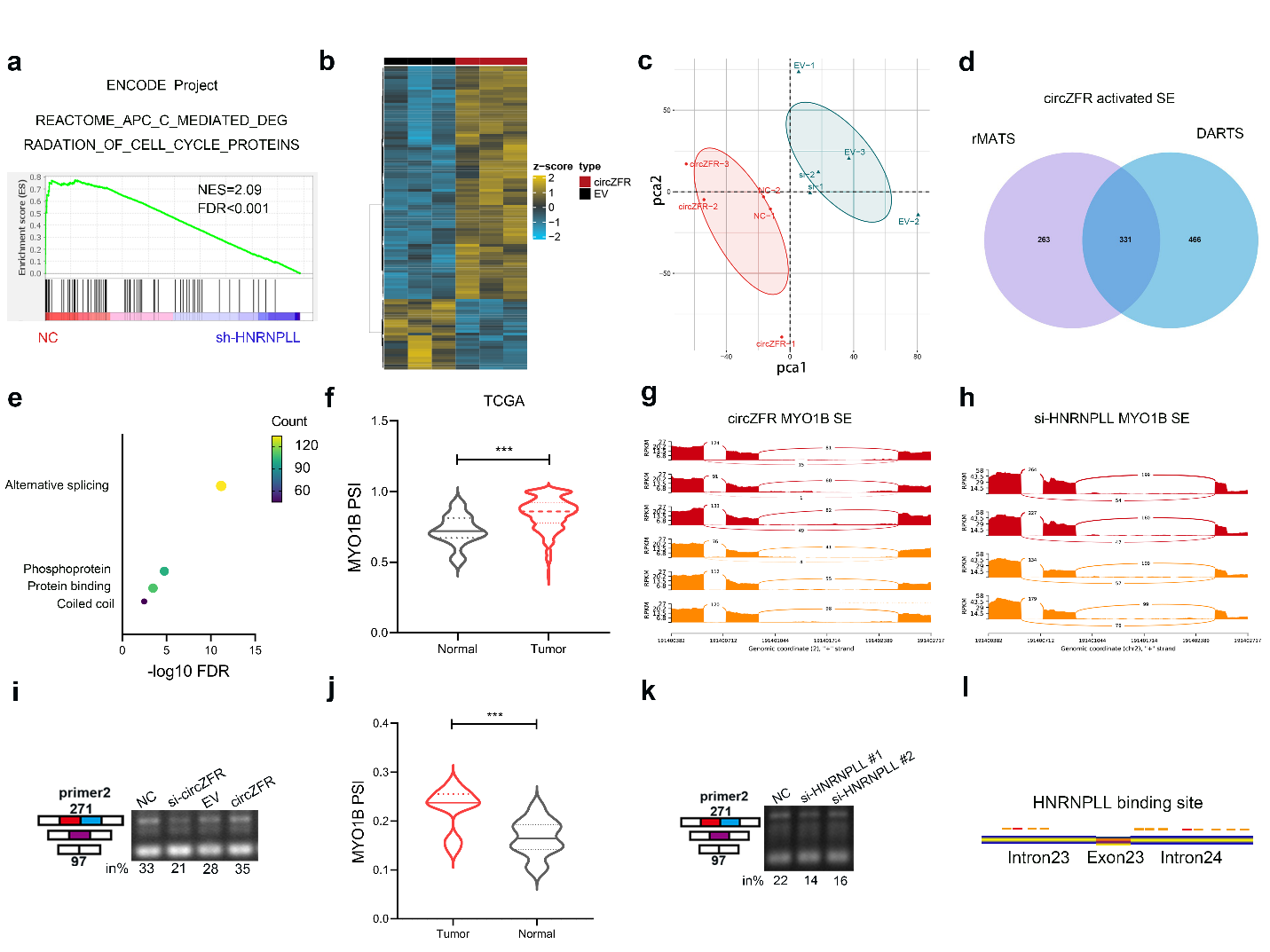


**Supplementary Figure 4. CircZFR regulates alternative splicing via HNRNPLL.**

**a** GSEA results of the differential genes affected by HNRNPLL knockdown in ENCODE project. **b** Heatmap of differentially expressed genes regulated by circZFR overexpression. **c** PCA of genes affected by circZFR overexpression and knockdown. Ellipses represent 95% confidence regions. **d** Pie chart showing skipped exons which were activated by circZFR and validated by deep leaning. **e** Gene Ontology analysis of both circZFR and HNRNPLL-sensitive targets analyzed by DAVID. **f** The inclusion of *MYO1B* exon 23 in TCGA dataset. **g, h** Sashimi plot of *MYO1B* exon inclusion affected by circZFR (**g**) and HNRNPLL (**h**). **i** RT-PCR validation of *MYO1B* exons in HCC827 cells. **j** Splicing pattern of *MYO1B* in LUAD and adjacent normal tissues as detected by RT-PCR. **k** The effects of HNRNPLL on *MYO1B* exons in HCC827 cells detected by RT-PCR. **l** Prediction of HNRNPLL binding sites on circZFR by CISBP-RNA. Data are shown as mean ± SD (n=3) or typical photographs of one representative experiment. *p < 0.05, **p < 0.01, ***p < 0.001, ns, nonsignificant, two-tailed Student’s t test.


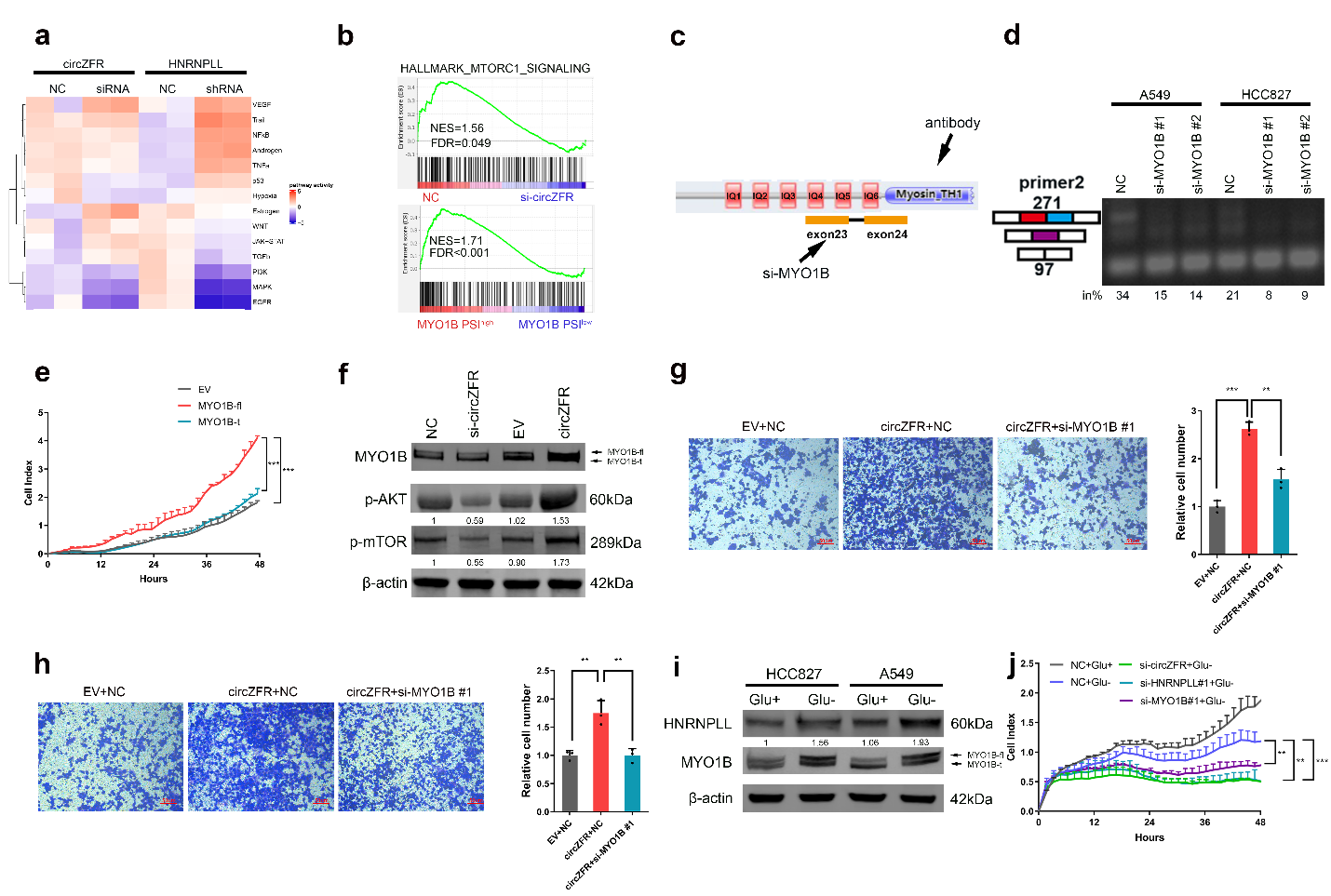


**Supplementary Figure 5. Alternative splicing of *MYO1B* is a functional target of circZFR.**

**a** Pathway activity regulated by circZFR and HNRNPLL calculated by PROGENy. **b** GSEA results of the differential genes affected by circZFR knockdown (upper panel) and MYO1B-fl expression (lower panel). **c** Schematic diagram of the siRNA and antibody targeting *MYO1B* splicing. **d** The effects of *MYO1B* siRNAs detected by RT-PCR. **e** The changes of cell vitality in A549 cells after transfecting with control or MYO1B vectors. **f** Western blot of the indicated proteins in the extracts of HCC827 cells. **g, h** Transwell (**g**) and matrigel (**h**) assays in A549 cells. Scale bars, 50 μm. **i** Western blot of the indicated proteins in HCC827 and A549 cells upon glucose starvation. **j** RTCA cell proliferation assays in A549 cells under glucose starvation. Cells were cultured in high (10mM) or low (2.5mM) glucose. Data are shown as mean ± SD (n=3). Similar results were obtained in three independent experiments or typical photographs of one representative experiment. *p < 0.05, **p < 0.01, ***p < 0.001, ANOVA followed by Tukey's test.


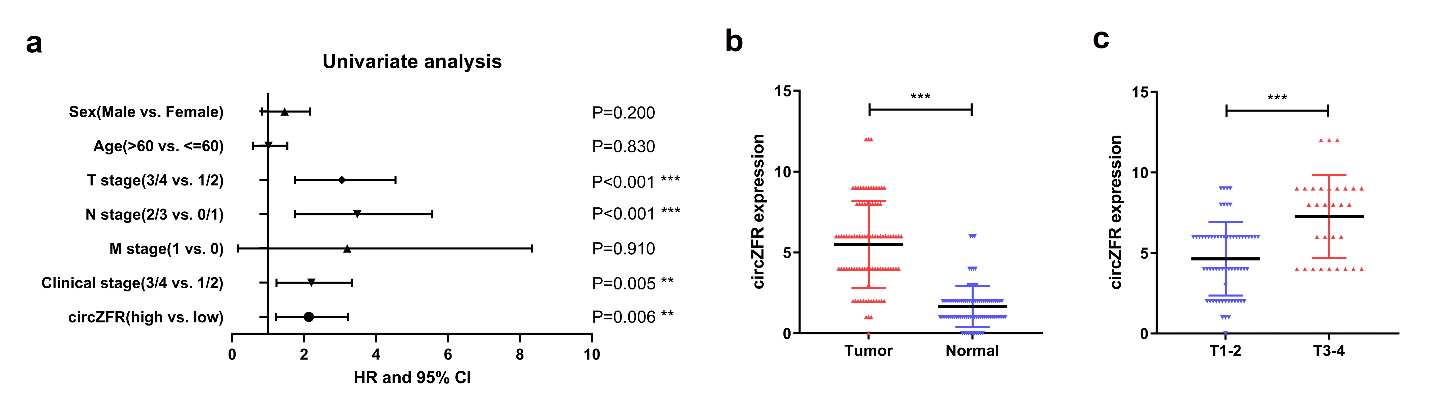


**Supplementary Figure 6. CircZFR is clinically relevant in LUAD.**

**a** Univariate analysis of circZFR in LUAD TMA. **b** The circZFR expression in LUAD and normal lung tissues detected by CISH. **c** The circZFR levels was correlated with T stage. Data are shown as mean ± SD. *p < 0.05, **p < 0.01, ***p < 0.001, two-tailed Student’s t test.
